# Dynamic in vitro intestinal barrier model coupled to chip-based liquid chromatography mass spectrometry for oral bioavailability studies

**DOI:** 10.1007/s00216-019-02336-6

**Published:** 2019-12-21

**Authors:** Milou J. C. Santbergen, Meike van der Zande, Arjen Gerssen, Hans Bouwmeester, Michel W. F. Nielen

**Affiliations:** 1 Laboratory of Organic Chemistry, Wageningen University, Stippeneng 4, 6708 WE Wageningen, The Netherlands; 2 TI-COAST, Science Park 904, 1098 XH Amsterdam, The Netherlands; 3 Wageningen Food Safety Research (WFSR), Wageningen University & Research, P.O. Box 230, 6700 AE Wageningen, The Netherlands; 4 Division of Toxicology, Wageningen University, Stippeneng 4, 6708 WE Wageningen, The Netherlands

**Keywords:** Gut-on-a-chip, Chip-based liquid chromatography, Mass spectrometry, Intestinal barrier, Oral bioavailability

## Abstract

In oral bioavailability studies, evaluation of the absorption and transport of drugs and food components across the intestinal barrier is crucial. Advances in the field of organ-on-a-chip technology have resulted in a dynamic gut-on-a-chip model that better mimics the in vivo microenvironment of the intestine. Despite a few recent integration attempts, ensuring a biologically relevant microenvironment while coupling with a fully online detection system still represents a major challenge. Herein, we designed an online technique to measure drug permeability and analyse unknown product formation across an intestinal epithelial layer of Caco-2 and HT29-MTX cells cultured on a flow-through Transwell system, while ensuring the quality and relevance of the biological model. Chip-based ultra-performance liquid chromatography quadrupole time-of-flight mass spectrometry (UPLC-QTOF-MS) was coupled to the dynamic Transwell system via a series of switching valves, thus allowing alternating measurements of the apical and basolateral sides of the in vitro model. Two trap columns were integrated for online sample pre-treatment and compatibility enhancement. Temporal analysis of the intestinal permeability was successfully demonstrated using verapamil as a model drug and ergotamine epimers as a model for natural toxins present in foods. Evidence was obtained that our newly developed dynamic system provided reliable results versus classical static in vitro models, and moreover, for the first time, epimer-specific transport is shown for ergotamine. Finally, initial experiments with the drug granisetron suggest that metabolic activity can be studied as well, thus highlighting the versatility of the bio-integrated online analysis system developed.

**Electronic supplementary material:**

The online version of this article (10.1007/s00216-019-02336-6) contains supplementary material, which is available to authorized users.

**Figure:**
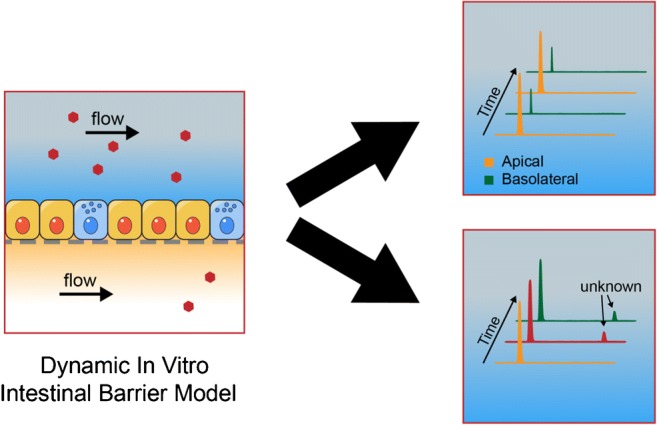
Graphical abstract

## Introduction

**Fig. 1:**
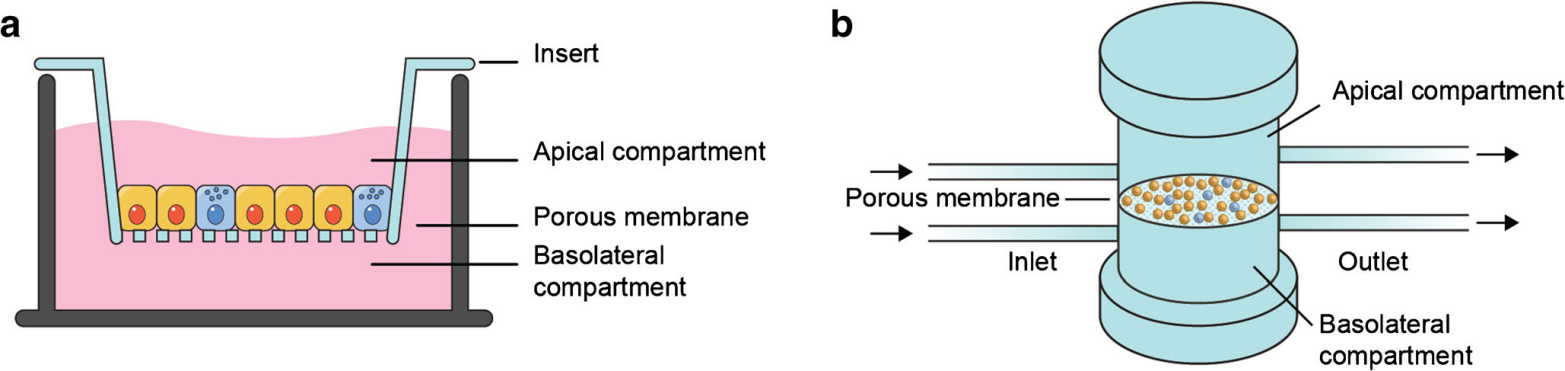
Schematic representation of (**a**) traditional Transwell insert and (**b**) dynamic flow-through Transwell system

To predict the in vivo bioavailability of drugs and dietary compounds, currently static in vitro cell culture models of the intestinal barrier are used. Often in these in vitro models, differentiated Caco-2 and HT29-MTX cells are used to model the intestinal epithelium [1]. Caco-2 cells are human intestinal epithelial cells which are able to form a tight polarized monolayer [2]. HT29-MTX cells are human mucin-producing goblet cells, depositing mucin on top of the cell barrier [3]. The incorporation of a mucin layer on the monolayer of cells renders an in vitro model that better mimics the in vivo intestinal barrier properties than a single Caco-2 cell model. These cells are generally cultured in a so-called Transwell system (Fig. 1a), which is a well-known system for permeability studies [4, 5]. Transwell systems are inserts containing a porous membrane on which monolayers of cells can be grown, creating an apical (lumen) and basolateral (bloodstream) compartment. A compound of interest is applied apically and allowed to permeate through the intestinal cell layer. Periodically, samples are taken from the apical and basolateral sides for measurement, to calculate the permeability of the compound. However, such a Transwell system has its limitations as the cells are grown in a static environment, thus ignoring the dynamic nature of the intestine.

Recent advances in organ-on-a-chip technology have led to state-of-the-art in vitro cell culture systems that include mechanical and functional features present in the human body, resulting in a much better representation of the in vivo microenvironment [6–8]. The gut-on-a-chip is an in vitro cell culture system that resembles the dynamic environment of the intestine. Several different geometries and designs have been developed for gut-on-a-chip devices, but two main types of designs are commonly found in the literature. The first one is based on the traditional Transwell system, where intestinal cells are grown on a circular porous membrane [9–12], and the second one is based on culturing cells on a porous membrane that separates two sides of a linear channel or tube [13–17]. Both types of systems contain an apical and basolateral flow of cell culture medium inducing shear forces on the cells and mimicking the luminal flow and blood flow. Dynamic flow systems not only feature a much better representation of the in vivo environment but also can reduce the use of cell culture media and bioreagents. Lastly, dynamic flow systems provide the possibility to fully automate the system by integration with an analytical detection system [18]. The latter, however, implies challenges for measurements, as the cell culture medium has a complex chemical composition that typically contains very high concentrations of salts, proteins, dyes and antibiotic drugs, which is not compatible with most analytical equipment.

Theoretically, electrospray ionization mass spectrometry (ESI-MS) would be the technique of choice for integration with a gut-on-a-chip device, as ionization takes place in the liquid phase. Furthermore, MS is well suited for multi-analyte detection, and apart from analyte transport across the cellular barrier, also unknown product formation (e.g. metabolites) can be monitored. However, due to the complex chemical composition of the cell culture medium, integration of dynamic in vitro models with ESI-MS requires extensive sample preparation in order to ensure system compatibility. Several semi-integrated systems have been reported, in which solid-phase extraction (SPE) columns were used as a sample pre-treatment step [19–23]. In these reports, the sample pre-treatment steps required manual operations to flush the SPE column, hampering full automation and compromising robustness of the system. Few online integrations have been described in the literature [24, 25], but unfortunately, they did not report on biological integrity for assuring a relevant organ-on-a-chip model. A complete online integration featuring an evidence-based biologically relevant dynamic in vitro model of the intestine and ESI-MS detection system has not been described yet in the literature.

In this study, we uniquely integrated a dynamic in vitro model of the intestine with a chip-based ultra-performance liquid chromatography quadrupole time-of-flight (UPLC-QTOF) MS analysis system for oral bioavailability and biotransformation studies. This combines a dynamic cell barrier system together with automated and continuous analytical readout of translocation processes and cell-induced product formation while maintaining full biological integrity and without compromising the analytical performance. For this, a co-culture of Caco-2 and HT29-MTX cells was grown in a flow-through Transwell system (Fig. 1b) and connected to the UPLC-QTOF-MS via a series of switching valves. The switching valves allowed for alternating UPLC-QTOF-MS measurements of the apical and basolateral sides of the flow-through Transwell system. Prior to chromatographic separation, two trapping columns were integrated in the series of switching valves for sample pre-treatment. To benchmark the online total analysis system for absorption studies, the permeability of two model compounds was examined (i.e. verapamil and ergotamine), in three different set-ups following a tiered approach. Firstly, permeability was evaluated in the traditional static Transwell system which allowed benchmarking against in vitro permeability data found in the literature. Secondly, permeability was evaluated in the flow-through Transwell system with offline measurements resulting in a one-to-one comparison between static and dynamic model systems. Thirdly, a truly automated online system was developed and compared to the offline flow-through Transwell version in order to assess the potential impact of its intrinsically higher complexity. Lastly, some initial experiments have been performed with the drug granisetron in order to show future application of the online analysis system for biotransformation studies.

## Experimental section

### Chemicals and reagents

Verapamil hydrochloride, ergotamin(in)e d-tartrate, penicillin-streptomycin, formic acid, Lucifer yellow, 4-(2-hydroxyethyl)-1-piperazineethanesulfonic acid (HEPES), sodium bicarbonate (NaHCO_3_), ammonium carbonate, Triton X-100 and Hank’s balanced salt solution (HBSS), with and without phenol red, were purchased from Sigma-Aldrich/Merck (Zwijndrecht, The Netherlands). Dulbecco’s Modified Eagle Medium (DMEM) with 4.5 g/L glucose and l-glutamine with and without phenol red, bovine serum albumin (BSA) and heat-inactivated foetal bovine serum (FBS) were purchased from Gibco (Bleiswijk, The Netherlands). Rabbit polyclonal antibody ZO-1/TJP1-Alexa Fluor 594, 4′,6-diamidino-2-phenylindole (DAPI), ProLong Diamond Antifade Mountant, dimethyl sulfoxide (DMSO), phosphate-buffered saline (PBS) and non-essential amino acids (NEAA) were obtained from Thermo Fisher Scientific (Landsmeer, The Netherlands). Acetonitrile and methanol were purchased from Actu-All Chemicals (Oss, The Netherlands). UPLC-MS grade water was purchased from Biosolve (Valkenswaard, The Netherlands). Granisetron was purchased from TCI Europe (Zwijndrecht, Belgium), paraformaldehyde from VWR (Amsterdam, The Netherlands) and water-soluble tetrazolium salt (WST-1) reagent from Roche Diagnostics GmbH (Mannheim, Germany), and water was prepared daily using a Milli-Q Reference Water Purification System from Millipore (Burlington, MA, USA).

### Cell culturing

The human colonic adenocarcinoma Caco-2 cell line was obtained from the American Type Culture Collection (ATCC, Manassas, VA, USA), and the human colon adenocarcinoma mucus-secreting HT29-MTX-E12 cell line was obtained from the European Collection of Authenticated Cell Cultures (ECACC, Salisbury, UK). Caco-2 cells were used at passage numbers 29–40, and HT29-MTX-E12 cells were used at passage numbers 52–70 for all experiments. Cells were cultured in DMEM containing 10% FBS, 1% NEAA and 1% penicillin-streptomycin and maintained at 37 °C in a 5% CO_2_-humidified air atmosphere and subcultured every 2 days to 3 days similar to the study of Abdelkhaliq et al. [26]. For permeability experiments, cells were seeded at a density of 40,000 cells/cm^2^ on a polycarbonate Transwell insert (area 0.6 cm^2^, 0.4 μm pore size; Millipore) as follows: Caco-2 and HT29-MTX-E12 cells were seeded in a 3:1 ratio on the apical side of the Transwell system and allowed to attach for 24 h. After 24 h, cell culture medium was refreshed every 2–3 days and Transwell systems were used for permeability experiments following 21 days of culturing.

### Evaluation of cell viability

Possible cytotoxic effects of verapamil, ergotamin(in)e and granisetron were evaluated using the cell proliferation WST-1 assay. With the WST-1 assay, mitochondrial activity of the cell is measured by the conversion of WST-1 into formazan dye by mitochondrial dehydrogenase enzymes. The assay is performed as follows: Caco-2 and HT29-MTX-E12 cells (ratio 3:1) were seeded in Greiner Bio-One (Alphen aan den Rijn, The Netherlands) flat-bottom 96-well plates at a concentration of 1 × 10^5^ cells/mL in cell culture medium (100 μL/well). Plates were incubated for 24 h at 37 °C under 5% CO_2_. Cell culture medium was removed, and subsequently, the cells were exposed to 100 μL/well serial dilutions of verapamil (0–500 μg/mL), ergotamin(in)e (0–20 μg/mL) or granisetron (0–1000 μg/mL) in cell culture medium for 24 h, at 37 °C. Then, the exposure medium containing (dilutions of) the compounds of interest was discarded and the cells were washed with pre-warmed HBSS. Subsequently, WST-1 reagent (in cell culture medium without phenol red) was added to the cells (1:10, 100 μL/well). After 1.5 h of incubation at 37 °C, the absorbance of each well was measured at 440 nm using a Bio-Tek (Winooski, VT, USA) Synergy HT Multi-Mode microplate reader. The viability of the cells for each concentration of verapamil and granisetron was expressed as a percentage of the negative control consisting of only cell culture medium. In the case of ergotamin(in)e, cell culture medium with 0.5% DMSO was used as a negative control, as ergotamin(in)e was dissolved in cell culture medium using 0.5% DMSO. Triton X-100 (0.25%, v/v) was used as a positive control in all cell viability assays and decreased the cell viability to 0.4% ± 0.3%.

### Evaluation of cell barrier integrity

Barrier integrity was evaluated similar to the study of Hubatsch et al. [27], and cells were cultured on a Transwell membrane for 21 days and subsequently stained for tight junction protein ZO-1 and the cell nuclei. For this, the cells were washed with PBS and fixed with 4% paraformaldehyde (w/v) for 15 min. Cells were permeabilized with 0.25% Triton X-100 (v/v) and blocked with 1% BSA (w/v). The cells were then incubated with the conjugated antibody ZO-1/TJP1-Alexa Fluor 594 for 45 min (10 μg/mL). Subsequently, DAPI was used to stain the nuclei for 10 min (5 μg/mL). Between incubations, cells were washed with PBS three times. Cells were mounted in a 120-μm spacer (Sigma-Aldrich) on a microscope slide (Thermo Scientific) with ProLong Diamond Antifade Mountant. Slides were then examined using a Zeiss (Jena, Germany) LSM 510 META confocal microscope. Samples were excited with 405-nm and 543-nm lasers, and the pinholes were in the range of 90–94 μm at a magnification of × 40. Cell layer integrity was also evaluated using the transport marker Lucifer yellow. Following permeability experiments, the cells were incubated with Lucifer yellow at a concentration of 500 μg/mL for 30 min on the apical side of the Transwell insert. Cell culture medium was collected from the apical and basolateral sides at *t* = 0 and *t* = 30 min and analysed for fluorescence at 458 nm/530 nm using a Bio-Tek Synergy HT Multi-Mode microplate reader. Cell layers that transported more than 5% of Lucifer yellow to the basolateral compartment were judged as leaking and were discarded.

### Cell permeability experiments using static and dynamic offline Transwell systems

For the static and dynamic offline cell permeability experiments, verapamil (5 μg/mL) and ergotamin(in)e (10 μg/mL) were used suspended in HBSS (without phenol red) containing 25 mM of HEPES and 0.35 g/L NaHCO_3_ as the donor solution. HEPES is added because the experiments with the flow-through Transwell systems are performed outside of a CO_2_-controlled incubator, and for the sake of comparability, it is added to the static Transwell systems as well. For the static Transwell experiments, these donor solutions were directly applied apically onto the cells (400 μL/insert), at day 21 of culturing. The basolateral side of the insert was filled with the same solution (600 μL/insert), but without the compound of interest (receiving solution). Samples (100 μL) were collected and replenished on the basolateral side at the following time points: 15 min, 30 min, 45 min, 60 min, 120 min and 180 min. Apical samples were taken at *t* = 0 min and *t* = 180 min. For the dynamic experiments, the Transwell inserts were placed into the QV600 system from Kirkstall (Rotherham, UK), further referred to as a flow-through Transwell system (see also Fig. 1b), at day 20 of culture. Cell culture medium containing 25 mM HEPES was perfused into the apical compartment (200 μL/min) and basolateral compartment (100 μL/min) of the flow-through Transwell system using a New Era Pump Systems (Farmingdale, NY, USA) syringe pump for 24 h at 37 °C, as described by Giusti et al. [28]. After 24 h, the apical syringe was replaced by a syringe containing the donor solution and the basolateral syringe was replaced by a syringe containing the receiving solution. In order to assure a biologically relevant environment, syringe heaters (New Era Pump Systems) were used to heat the medium and keep the cells at 37 °C without the need for an additional incubator. Both effluent flows were attached to a Gilson 234 autosampler (Villiers-le-Bel, France) which was used as a fraction collector, collecting samples every 2 min in 96-well plates. All experiments were conducted in biological triplicates.

### Offline targeted LC-MS/MS analysis

#### Verapamil

Verapamil static and dynamic offline Transwell samples were diluted 500 times in water prior to LC-MS/MS analysis. Chromatographic separation was achieved using a Waters (Milford, MA, USA) Acquity I Class UPLC system equipped with an Acquity UPLC BEH C18 (100 mm × 2.1 mm, 1.7 μm) column (Waters). The column temperature was kept at 50 °C, and the autosampler was set at 10 °C. A 2.5 μL injection volume was used. Mobile phase A was water, and mobile phase B was acetonitrile, both containing 5 mM formic acid. The mobile phase gradient started at 10% B and, after 0.8 min, was linearly increased to 40% B in 2 min followed by an increase to 99% B in 0.1 min. This composition was kept for 3 min and returned to 10% B in 0.1 min, all at a constant flow rate of 0.6 mL/min. A mobile phase equilibration time of 1.1 min was allowed prior to the next injection. The first minute of the gradient elution was directed to waste in order to prevent any salts from the HBSS matrix to enter the ionization source. Mass spectrometric detection was performed using a Waters Xevo TQS tandem mass spectrometer equipped with an electrospray ionization interface (ESI) and operated in positive ion mode, with a capillary voltage of 3.1 kV, a desolvation temperature of 450 °C, a gas flow rate of 800 L/h, a source temperature of 150 °C and a cone gas flow rate of 150 L/h. For verapamil, three multiple reaction monitoring (MRM) transitions were acquired (Table S1 in the Electronic Supplementary Material, ESM). For quantification of verapamil, a calibration curve was constructed. Calibration solutions were prepared in HBSS and subsequently diluted in water similar as the samples, resulting in a calibration curve with the following points: 0 μg/mL, 0.01 μg/mL, 0.05 μg/mL, 0.1 μg/mL, 0.25 μg/mL, 0.5 μg/mL, 1 μg/mL and 5 μg/mL. Limit of detection (LOD) and limit of quantification (LOQ) were calculated as the average signal of five blank samples plus three (LOD) or ten (LOQ) times the standard deviation (SD) of the blank samples divided by the sensitivity (slope of the calibration curve) using the transition m/z 455.4 > 165.1, resulting in a LOD of 3.3 ng/mL and a LOQ of 9.6 ng/mL. The concentration of verapamil in the unknown samples was then calculated using peak area measurements in the reconstructed ion current (RIC) of the respective MRM transitions via interpolation of the external matrix-matched calibration curve (*r*^2^ = 0.99).

#### Ergotamin(in)e

Apical and basolateral ergotamin(in)e samples of the static Transwell experiments were diluted 20 times in methanol/water 60:40 (v/v) ratio prior to LC-MS/MS analysis, as were the apical Transwell samples of the dynamic experiments. The basolateral samples of the dynamic experiments were analysed undiluted. LC-MS/MS analysis was performed as described for verapamil with the following modifications: a 2 μL injection volume was used. Mobile phase A consisted of 10 mM ammonium carbonate in water (pH 9), and mobile phase B consisted of acetonitrile. The mobile phase gradient started at 0% B and was linearly increased to 10% B in 1 min followed by an increase to 30% B in 10 min and returned to 0% B in 0.2 min, all at a constant flow rate of 0.4 mL/min. A mobile phase equilibration time of 2.6 min was allowed prior to the next injection. The first minute of the gradient elution was directed to waste to prevent salts from the HBSS matrix to enter the ion source of the MS. Mass spectrometric detection was performed as described for verapamil with the following modifications: the mass spectrometer was operated in positive ion mode with a capillary voltage of 3.0 kV and a desolvation temperature of 600 °C. For ergotamin(in)e, four MRM transitions were measured (ESM Table S1). For quantification of ergotamin(in)e, a calibration curve was constructed. Calibration solutions were prepared in HBSS and subsequently diluted in methanol/water 60:40 (v/v) ratio similar as the samples, resulting in a calibration curve with the following points: 0 μg/mL, 0.01 μg/mL, 0.05 μg/mL, 0.1 μg/mL, 0.5 μg/mL, 1 μg/mL, 2.5 μg/mL, 5 μg/mL and 10 μg/mL. LOD and LOQ were calculated as the average signal of five blank samples plus three (LOD) or ten (LOQ) times the SD of the blank samples divided by the sensitivity (slope of the calibration curve) using the transition m/z 582.4 > 208.1, resulting in a LOD of 1 ng/mL and a LOQ of 1.9 ng/mL. The concentration of ergotamin(in)e in the unknown samples was then calculated using the peak area measurements in the RIC of the respective MRM transitions via interpolation of the external matrix-matched calibration curve (*r*^2^ = 0.99).

### Flow-through Transwell system coupled online with chip-based UPLC-QTOF-MS

**Fig. 2:**
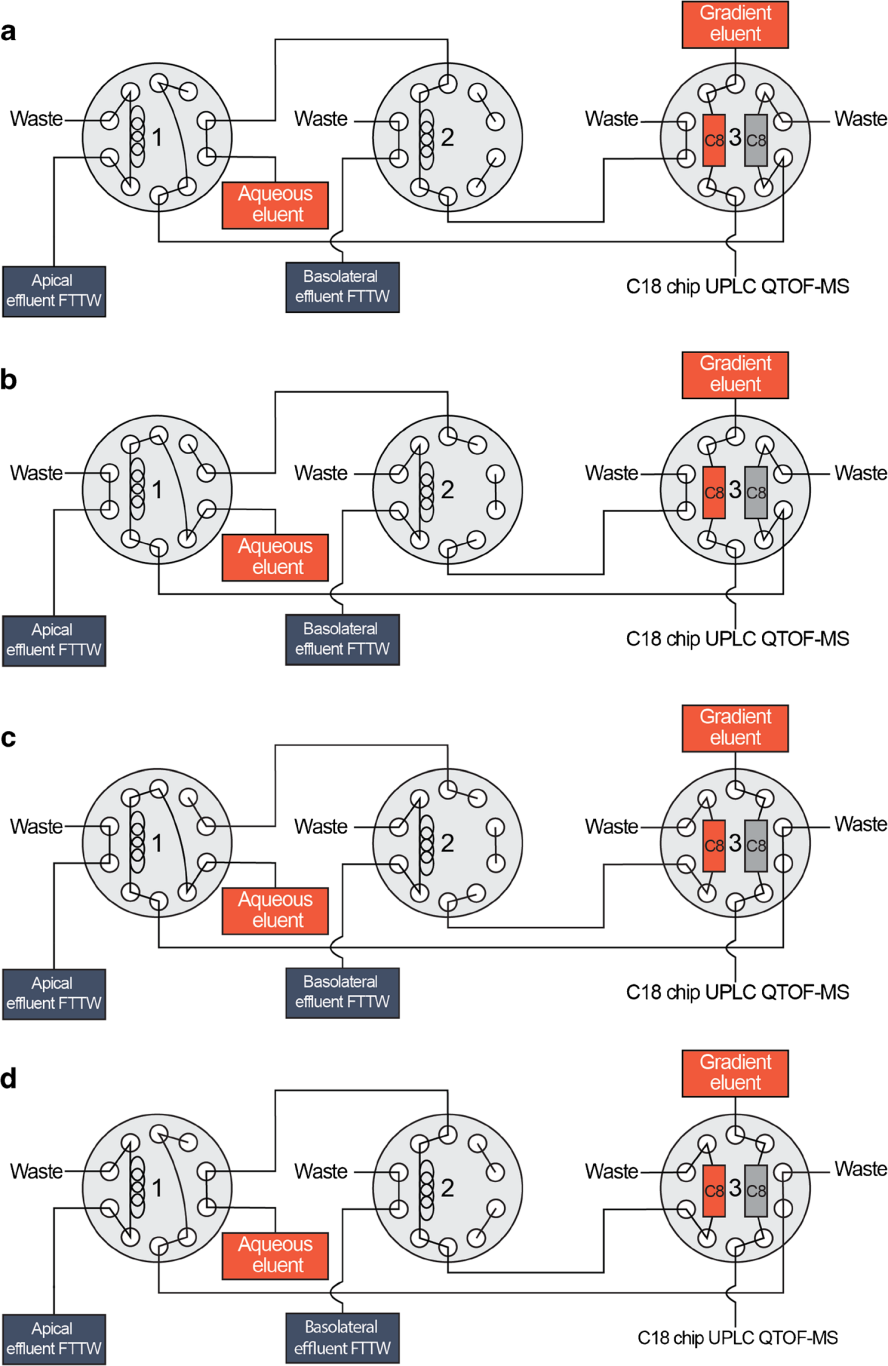
Schematic representation of the flow-through Transwell (FTTW) system coupled to chip-based UPLC-QTOF-MS using parallel traps as interface. The set-up consists of three switching valves, two sample loops, two C8 trap columns and a C18 chip analytical column. (**a**) Sample effluent from the apical side of the FTTW system fills the sample loop in the first switching valve for 15 min, while an aqueous effluent flows through the second sample loop to the waste. (**b**) After the 15-min sample collection, the first valve switches and aqueous effluent flows through the first sample loop towards the C8 trap column in the third switching valve. The aqueous effluent loads the sample onto the trap column and rinses away the salts. At the same time, the second valve switches to collect effluent from the basolateral side of the FTTW system. (**c**) Subsequently, a gradient from aqueous to organic solvent runs through the trap column in the third valve, through the analytical column and to the MS. (**d**) The next valve switch flushes the sample collected from the basolateral side of the FTTW system to the trap column. The analytes captured on the trap column are eluted by the gradient from aqueous to organic solvent onto the analytical column and to the MS (**a**). Total analysis from the trap column to the analytical column takes 15 min. The sequence repeats until the entire dynamic in vitro biological experiment is completed

All dynamic online cellular experiments were conducted using the flow-through Transwell system (Fig. 1b). A series of three 2-position/10-port UltraLife switching valves (IDEX Health & Science, Oak Harbor, WA, USA) with 1/16″ fittings were used to interface the flow-through Transwell system to the chip-based UPLC-QTOF-MS. Apical and basolateral effluents from the flow-through Transwell system were alternatingly loaded onto 5-μL stainless steel sample loops, mounted on the first and second switching valves (Fig. 2). Each sample loop was loaded for 15 min at 200 μL/min (apical) and 100 μL/min (basolateral), and the detailed valve switching program is listed in Table S2 (ESM). After 15 min of sample collection, the sample loop was flushed for 4 min towards an Optimize Technologies (Oregon City, OR, USA) C8 trapping column (180 μm × 5 mm, 2.7 μm) using an aqueous solvent (H_2_O with 1% acetonitrile) at a flow rate of 20 μL/min. Following the clean-up, the trap column was eluted towards an iKey Chip BEH C18 analytical column (150 μm × 50 mm, 1.7 μm) (Waters). The 3 μL/min microflow gradient for verapamil and granisetron experiments consisted of mobile phase A (water with 1% acetonitrile) and mobile phase B (acetonitrile with 1% water), both containing 0.1% formic acid. The gradient started at 0% B and, after 4 min, was linearly increased to 100% B in 4 min. This composition was kept for 3 min and returned to 0% B in 0.1 min. An equilibration time of 3.9 min was allowed prior to the next injection. For ergotamin(in)e experiments, the gradient consisted of mobile phase A (water with 10 mM ammonium carbonate) (pH 9) and mobile phase B (acetonitrile). The gradient started at 30% B and, after 4 min, was linearly increased to 70% B in 6 min. This composition was kept for 1 min and returned to 30% B in 0.2 min. An equilibration time of 3.8 min was allowed prior to the next injection. The complete process is illustrated in Fig. 2. Data were collected using MassLynx v4.1 software, yielding a separate data file for each trap column analysis or, in other words, alternatingly, for the apical and basolateral sides of the dynamic in vitro model. For quantification, the peak area of the RIC of the basolateral measurement was relative to the average peak area of the RIC in the apical sample measurements. Mass spectrometric detection was performed with a Waters ionKey Xevo QTOF MS system. The mass spectrometer was operated in positive ion mode, with a capillary voltage of 3.9 kV, a desolvation temperature of 350 °C, a gas flow rate of 400 L/h, a source temperature of 80 °C and a cone gas flow rate of 10 L/h.

### Permeability calculations

The apparent permeability coefficient (*P*_app_, cm/s) was calculated as described by Yeon and Park [29], according to the following equation:

$$ {P}_{\mathrm{app}}=\frac{\mathrm{d}Q}{\mathrm{d}t}\frac{1}{A\ {C}_0} $$

where d*Q*/d*t* is the cumulative transport rate in the basolateral compartment (μmol/s), *A* is the surface area of the cell layer (0.6 cm^2^) and *C*_0_ is the initial concentration of the compounds in the apical compartment (μmol/cm^3^).

## Results and discussion

### Evidence-based bio-integrity of the intestinal barrier

**Fig. 3:**
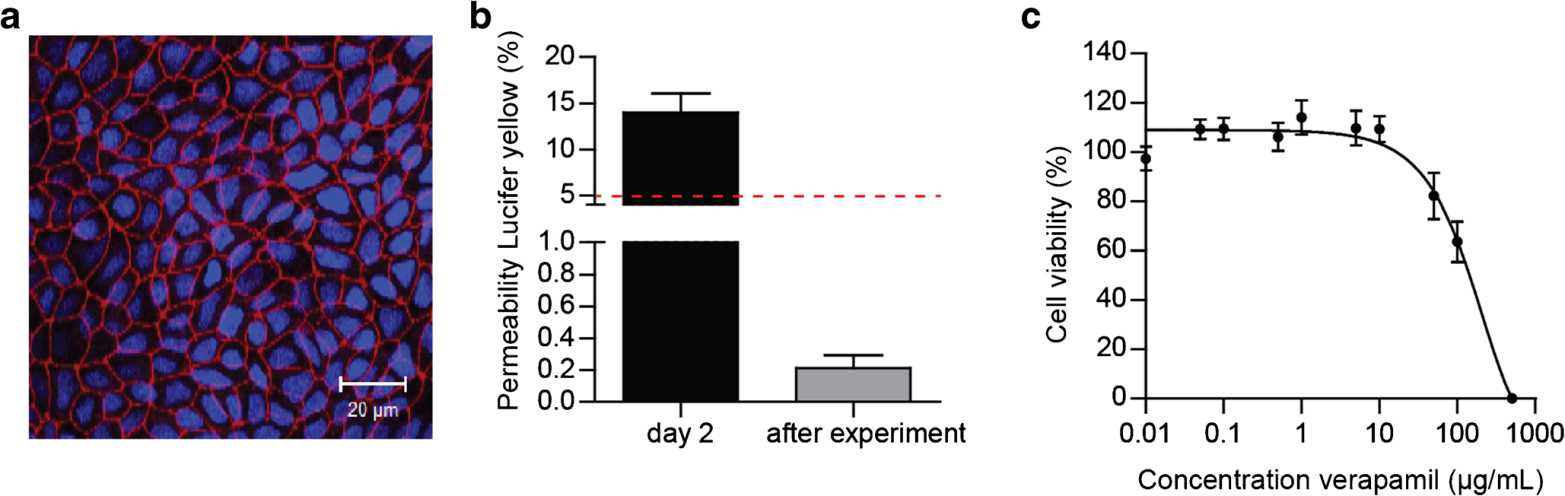
(**a**) Confocal image of Caco-2/HT29-MTX cells cultured in a Transwell system for 21 days. Cells were stained for tight junction protein ZO-1/TJP1 (red) and cell nuclei (blue). (**b**) Permeability of Lucifer yellow across a monolayer of Caco-2/HT29-MTX cells after 2 days of culture and after permeability experiments (day 21), given as a percentage of the apical concentration (% ± standard error of the mean (SEM)). (**c**) Cell viability of a Caco-2/HT29-MTX-E12 co-culture after the 24-h exposure to increasing concentrations of verapamil using the WST-1 mitochondrial activity assay. Viability is given as a percentage of the control (% ± SEM; *n* = 3)

The human gastrointestinal tract is the second largest organ in the human body. It protects our body from pathogens and toxic compounds. On the other hand, it effectively absorbs nutrients from our food as well as orally administrated drugs. A reliable in vitro model should reflect this gatekeeper function. A prerequisite for in vitro permeability experiments is the formation of a leak-free monolayer of intestinal cells grown on a permeable membrane (i.e. Transwell). In our in vitro model, confocal imaging showed a network of tight junctions (Fig. 3a), demonstrating a tight monolayer of cells after 21 days of culture in the Transwell system. Furthermore, Lucifer yellow permeability testing following each compound permeability experiment indicated good barrier integrity of the monolayer, as Lucifer yellow is a marker for paracellular transport. Less than 5% Lucifer yellow permeability is generally used as a cut-off value for a leaky monolayer [30], and an average permeability of 0.18% ± 0.06% was experimentally obtained (Fig. 3b). To assess the flow-through Transwell model as a model for oral absorption, we examined its permeability characteristics using the model compound verapamil, of which extensive in vitro and in vivo permeability data can be found in the literature. Prior to the permeability experiments with verapamil, a non-toxic concentration of verapamil was determined to ensure proper cell viability during experiments. For this, we incubated non-differentiated cells for 24 h with increasing concentrations of verapamil to establish the desired non-toxic concentration for subsequent studies. These are worst-case conditions as in the permeability experiments, the exposure lasted 3 h and was executed on fully differentiated cells. The combination of a longer exposure time and the use of proliferating cells results in a higher sensitivity towards cytotoxicity, compared to the shorter incubation and fully differentiated cells used in permeability experiments. The results showed that the cell viability of the co-culture of Caco-2 and HT29-MTX-E12 cells was > 80% after exposure to concentrations of verapamil at or below 10 μg/mL (Fig. 3c). Therefore, a concentration of 5 μg/mL was selected for permeability experiments in both the static and dynamic model systems.

### Online flow-through Transwell analysis system, design and performance

Initial designs of the online total analysis system consisted of different configurations of switching valves (ESM Fig. S1), providing shorter run times and the possibility to select which effluent to measure. However, these configurations experienced severe analyte carryover between the two effluent streams, due to shared capillary tubing (carryover experiments described in the ESM). Finally, the in vitro model of the human intestinal epithelium could be successfully integrated with chip-based UPLC-QTOF-MS, using the set-up as shown in Fig. 2, thanks to the separate tubing and traps for apical and basolateral effluents, having high and low concentrations of analytes, respectively. A flow-through Transwell system (Fig. 1b) containing a co-culture of Caco-2 and HT29-MTX cells was operated upstream by a syringe pump equipped with a syringe heater to maintain a physiologically relevant temperature of the cell culture medium. HEPES was added to the cell culture medium as a pH buffer to maintain the physiologically relevant microenvironment. After starting the exposure for 15 min, apical effluent was collected in the sample loop of the first switching valve (Fig. 2a). The sample was subsequently directed to a C8 trap column integrated on the third switching valve, allowing for analyte trapping and desalting (Fig. 2b). After trapping, the analyte was separated on a chip-based reversed-phase C18 column having an integrated nano-electrospray ionization emitter and analysed by QTOF-MS (Fig. 2c). The total analysis time including sample trapping, desalting and MS measurement was 15 min. During the 15-min time period, the sample loop in the second switching valve was filled with the basolateral effluent for the next measurement (Fig. 2b, c) following the same trapping and desalting steps (Fig. 2d), but in a fully independent fluidic part of the set-up in order to exclude any carryover. Thus, every 15 min, there was a switch between the apical and basolateral channels and this process was repeated until the end of the biological experiment. This final design turned out to be the best compromise with regard to lack of carryover, analysis time, frequency of sampling and analytical robustness. System stability was proven by introducing 5 μg/mL verapamil in HBSS for 3 h into the automated total analysis system by a syringe pump. During the complete run time of the experiment, the retention time for the verapamil peak was stable at 6.8 min (ESM Fig. S2a). Also, the pressure profile of the UPLC gradient pump attached to the third switching valve that runs over the trap columns and the analytical column confirmed no increase (column clogging) or decrease of pressure during the 3-h measurements (ESM Fig. S2b), thus demonstrating adequate removal of the very high level of salts in the medium.

### Temporal analysis of intestinal permeability

#### Verapamil

**Fig. 4:**
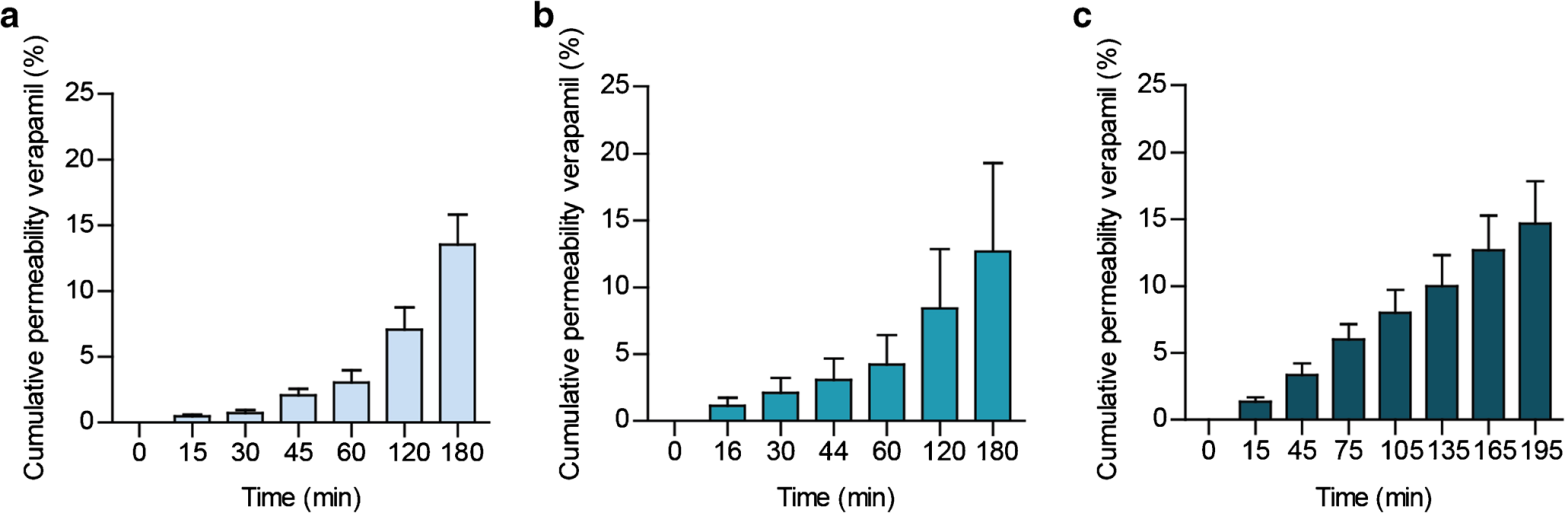
(**a**) Permeability of verapamil across a monolayer of Caco-2/HT29-MTX cells in a static Transwell system. Permeability is given as a percentage of the apical concentration (% ± SEM; *n* = 3). (**b**) Permeability of verapamil across a monolayer of Caco-2/HT29-MTX cells in the dynamic flow-through Transwell system. Permeability is given as a calculated cumulative percentage of the apical concentration (% ± SEM; *n* = 3). (**c**) Permeability of verapamil across a monolayer of Caco-2/HT29-MTX cells in the dynamic flow-through Transwell system with online analysis. Permeability is given as a calculated cumulative percentage of the apical concentration (% ± SEM; *n* = 3)

Verapamil (ESM Fig. S3) was selected as a model compound as it is classified in category II of the Biopharmaceutics Classification System (BCS), meaning it is a high-permeability compound, and it is known to cross the intestinal epithelium predominantly via passive transcellular diffusion [31, 32]. In order to assess the flow-through Transwell model as a model for oral absorption and to benchmark the automated online analysis system, three different set-ups were compared for verapamil permeability: (1) the traditional static Transwell system, (2) the dynamic flow-through Transwell system with offline sample collection and (3) the dynamic flow-through Transwell system with online analysis. For the static experiment, samples were collected on the basolateral side of the membrane at *t* = 0 min, 15 min, 30 min, 45 min, 60 min, 120 min and 180 min. In the dynamic offline system, samples were collected every 2 min using a fraction collector in a 96-well plate. The concentration of verapamil was determined by LC-MS/MS. Cumulative transport of verapamil across the intestinal layer in the static and dynamic offline system after 3 h was 13.5% and 12.7% of the exposure concentration, respectively, and followed a similar trend over time (Fig. 4a, b). Variation among the biological samples in the dynamic system was somewhat larger, compared to the static system, but note that in this work, biological triplicate experiments were performed so all data points include both variations in cell culturing, in permeability and the analytical variation. The difference in variation in the dynamic system is probably due to some flow-induced shear stresses on the cells [33]. Having established that the permeability of verapamil is similar in a static and dynamic environment, thereby confirming a biologically functional in vitro intestinal barrier in the dynamic system, we used the verapamil permeability data from the dynamic offline system to benchmark the dynamic online analysis system. The cumulative transport data from the fully integrated online analysis system clearly demonstrates the accuracy and robustness of the automated system, as the results showed high similarity with the dynamic offline system, with a transport of 15.0% after 3 h of exposure (Fig. 4c). Additionally, the apparent permeability coefficients were calculated for all verapamil experiments, allowing comparison of our results against static in vitro permeability data found in the literature (Table 1). As shown in Table 1, the apparent permeability coefficients observed in our study corresponds with the range of data found in the literature. Comparison of our dynamic automated system with other dynamic systems is challenging as there is a high variety in designs and there are only a limited number of studies examining the absorptive capacity of the model [10, 14, 20, 40, 41]. For a widespread use of dynamic in vitro intestinal barrier models and incorporation of them in drug development trials and risk assessment approaches, more permeability data of model compounds needs to be generated using the dynamic devices, including benchmarking against the static Transwell and in vivo data.

**Table 1:** Apparent permeability coefficient of verapamil described in the literature for different static in vitro model systems versus data obtained in this study

Cell model	*P*_app_ × 10^−6^ cm/s	Ref.
Caco-2	9.17–62.4	[34–37]
Caco-2 (TC-7)^a^	2.98 ± 0.55	[38]
Caco-2/HT29-MTX	41.7 ± 4.7	[39]
Caco-2/HT29-MTX/Raji B^b^	42.7 ± 5.6	[39]
LLC-PK1^c^	24 ± 15.3	[34]
L-MDR1^c^	18.8 ± 0.7	[34]
Caco-2/HT29-MTX (static)	20.9 ± 3.9	This study
Caco-2/HT29-MTX (dynamic)	19.5 ± 10.3	This study
Caco-2/HT29-MTX (dynamic online)	20.8 ± 4.3	This study

^a^TC-7 is a clone of the Caco-2 cell line

^b^Raji B is a human B lymphocyte cell line

^c^LLC-PK1 cells are porcine epithelial cells, and L-MDR1 (multidrug-resistant) cells are LLC-PK1 cells stably transfected with human MDR1 cDNA

#### Ergotamin(in)e

**Fig. 5:**
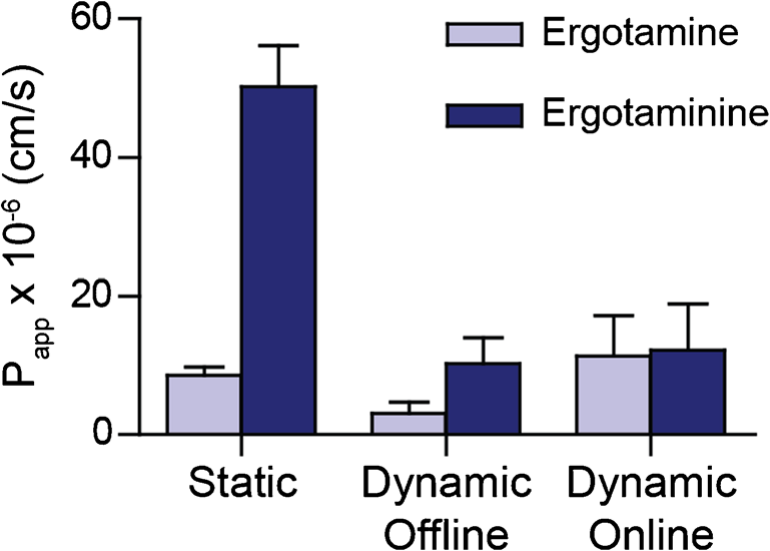
*P*_app_ values of ergotamine and ergotaminine obtained in the static and dynamic (offline and online) systems (± SEM, *n* = 3)

Apart from evaluating the permeability of the widely studied drug verapamil, we also examined the permeability of the epimers ergotamine and ergotaminine (ESM Fig. S4), from the ergot alkaloid family, across the intestinal barrier. Evaluating the oral bioavailability of ergot alkaloids is highly relevant as they can contaminate food and feed and little is known about their absorption characteristics or transport mechanisms [42]. Ergot alkaloids are found in plants infected with the fungus *Claviceps purpurea*. High oral intake of ergot alkaloids via contaminated food can result in ergotism also known as Saint Anthony’s fire, with symptoms such as headaches, spasms and vasoconstriction [42]. Even though the –inine form of ergot alkaloids is considered not biologically active [43], epimerization can occur under various conditions and could thus add to toxicity [44]. Just like for verapamil, we first established a non-toxic concentration for ergotamin(in)e as a mixture. As shown in Fig. S5 (ESM), the non-toxic concentration for ergotamin(in)e was selected at 10 μg/mL and lower. Human studies have shown overall low oral bioavailability of ergotamine [45, 46], and it has been classified to be a BCS III compound, meaning a low-permeability compound [47]. Therefore, we used the highest non-toxic concentration possible for the ergotamin(in)e permeability experiments (10 μg/mL). The apparent permeability coefficient of ergotamine across the barrier of Caco-2 and HT29-MTX cells in the static experiments was 8.6 ± 1.2 × 10^−6^ cm/s. Interestingly, the apparent permeability coefficient of the ergotaminine epimer was 50.2 ± 5.8 × 10^−6^ cm/s (Fig. 5). Higher-permeability measurements of ergotaminine compared to ergotamine could be due to epimerization of ergotamine to ergotaminine; however, this is chemically rather unlikely as the microenvironment conditions are kept stable during the 3-h experiments with no changes in pH or temperature. Another explanation could be that ergotamin(in)e is actively transported across the intestinal membrane and the transporter has a higher affinity for the –inine form. The opposite can also be true that an efflux transporter like P-glycoprotein (P-gp) has more affinity with ergotamine than with ergotaminine. Bromocriptine, another member of the ergot alkaloid family, is a known substrate for the efflux transporter P-gp [48]. For verapamil and ergotamine, we found similar permeability coefficients across the static and dynamic model systems, whereas for ergotaminine, permeability is 5 times lower in the dynamic experiments compared to the static experiments. Interestingly, in the dynamic experimental (both offline and online) measurements, the large difference between the epimer permeability rates is lost (Fig. 5). *P*_app_ values for the ergotaminine epimer in the dynamic experiments are on the same level as for the ergotamine epimer. This difference clearly suggests that not the microenvironment as such but the cell layer is responsible for selective transport of the epimers and/or epimerization. Apparently, cell-induced epimerization and/or active epimer selective transport is highly influenced by shear stress, highlighting the importance of dynamic systems for accurate representation of the microenvironment.

### Potential for online analysis of cell-induced product formation

**Fig. 6:**
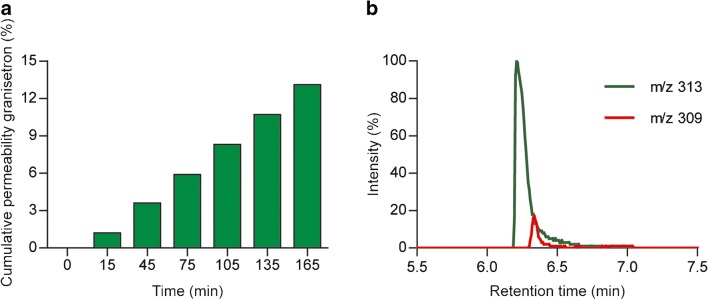
(**a**) Permeability of granisetron across a monolayer of Caco-2/HT29-MTX cells in the dynamic flow-through Transwell system with online analysis. Permeability is given as a calculated cumulative percentage of the apical concentration (*n* = 1). (**b**) reconstructed ion chromatogram of m/z 313 ([M+H]^+^) and 309 (unknown) at the time point of 30 min on the apical side

With the analysis of verapamil and ergotamin(in)e, we have demonstrated the robustness of the online analysis system to measure intestinal absorption in time. Besides temporal permeability analysis, the system could be suited for the detection of cell-induced product formation as well. For an initial feasibility experiment, we selected the compound granisetron (ESM Fig. S6), which is a serotonin 5-HT_3_ receptor antagonist as a model substrate for metabolism in our intestinal barrier model. Granisetron is an oral drug that is used against nausea as a result of chemotherapy [49] and is metabolized by the enzymes CYP1A1 and CYP3A4, resulting in 7-hydroxygranisetron and 9-desmethylgranisetron, respectively [50]. Caco-2 cells express the CYP1A1 enzyme, suggesting that granisetron might be metabolised in our Caco-2/HT29-MTX model [51]. Again first, a non-toxic concentration of 10 μg/ml granisetron was established (ESM Fig. S7). Next, an initial experiment was conducted using the newly developed dynamic online analysis system. After 3 h of exposure, 13.1% of granisetron translocated the in vitro intestinal barrier (Fig. 6a), but unfortunately, no cell-induced products were detected on the basolateral side of the cell barrier. Nevertheless, an additional ion with m/z 309 appeared on the apical side of the membrane (Fig. 6b). It was confirmed that the formation of this unknown compound was due to the presence of the cells, as the ion at m/z 309 was not detected after the 24-h incubation in the same matrix at 37 °C without cells (ESM Fig. S8). In other words, we can exclude chemical degradation as the cause of the product formation and, therefore, the formation of this unknown compound is due to the biologically active cells through either metabolism of granisetron or excretion of an unknown cell metabolite. However, more research is needed to elucidate the structure of this compound. Nevertheless, these results demonstrate the applicability and robustness of the automated online analysis system for measuring unknown product formation in the in vitro intestinal model.

## Conclusions

Here, we developed an integrated online analysis system for the evaluation of intestinal absorption and unknown product formation by using a flow-through Transwell system and mass spectrometry, while ensuring full bio-integrity of the in vitro intestinal barrier. The biggest strength of the online analysis system is the combination of a functional dynamic biological model of the intestine with semi-continuous online analytical detection. The permeability of verapamil and ergotamin(in)e across a monolayer of Caco-2 and HT29-MTX cells was successfully studied in a time-resolved manner without any manual handling. Furthermore, we were able to detect unknown product formation upon exposing the intestinal cell layer to granisetron. While this work should be considered as a proof of concept, a wider range of different drugs, toxins or dietary compounds can be easily evaluated using this system. The system even allows for incorporation of other cell types in the flow-through Transwell system for more complex biological mechanistic-based studies. Last but not least, the system also allows future extensions with a dynamic in vitro oral digestion system or a liver compartment in order to provide a full oral bioavailability system with semi-continuous readout of absorption and biotransformation.

## Electronic supplementary material

ESM 1 (PDF 559 kb)
