# Supplementary material for: Dynamic in vitro intestinal barrier model coupled to chip-based liquid chromatography mass spectrometry for oral bioavailability studies
Source: Anal Bioanal Chem. 2019 Dec 21;412(5):1111–22. doi: 10.1007/s00216-019-02336-6 (PMC7007416; doi:10.1007/s00216-019-02336-6)
Supplement: Supplementary file 1 — (PDF 559 kb) [file 216_2019_2336_MOESM1_ESM.pdf]

Analytical and Bioanalytical Chemistry

Electronic Supplementary Material

Dynamic in vitro intestinal barrier model coupled to chip-based liquid chromatography mass spectrometry for oral bioavailability studies

Milou J.C. Santbergen, Meike van der Zande, Arjen Gerssen, Hans Bouwmeester, Michel W.F. Nielen

Supplemental Figures

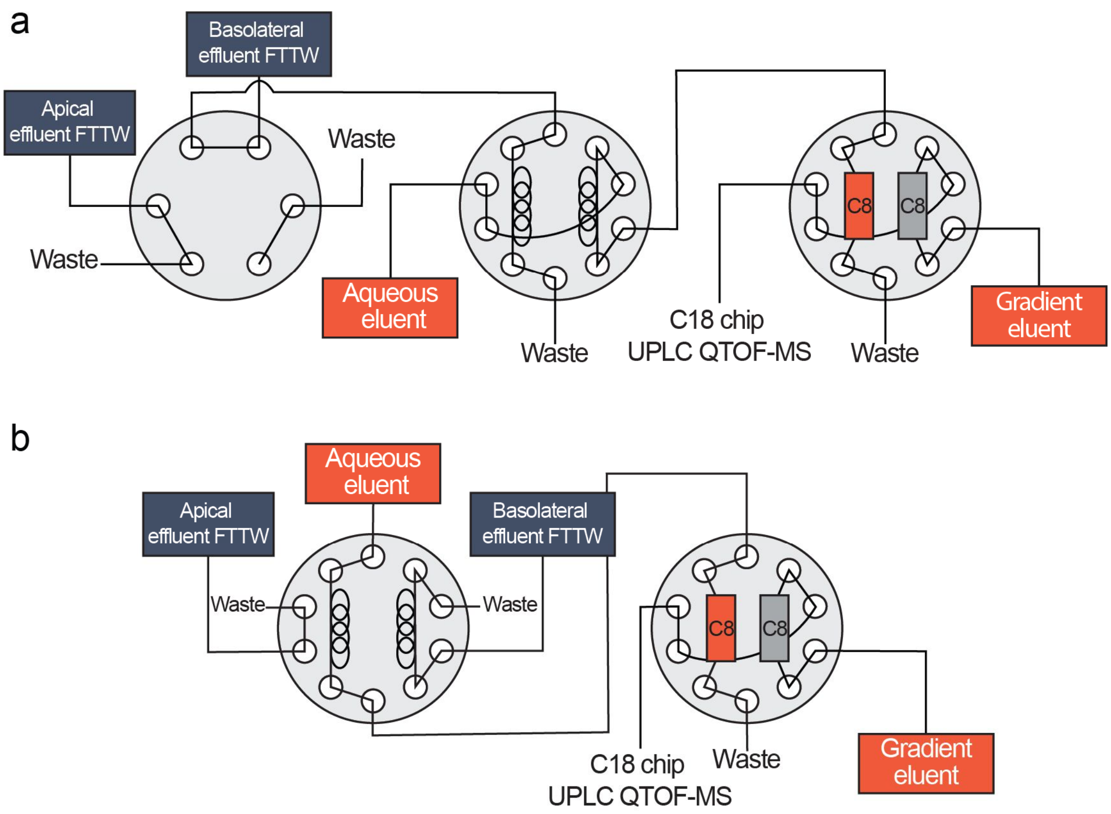

Fig. S1 Initial configurations of the switching valves. **a** Series of three switching valves with the possibility to choose the effluent stream to be measured in the first valve, sample collection in the second valve and analyte trapping in the third valve. **b** Two switching valves, for alternating apical and basolateral sample analysis. In the first valve sample was collected and in the second valve the analyte was trapped on a trap column

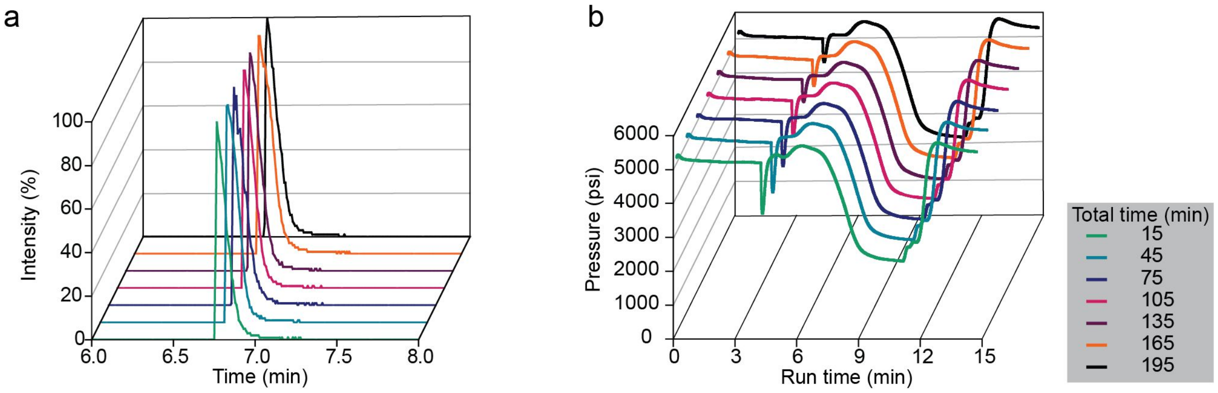

Fig. S2 a Peak stability of verapamil during three hours of measurements using the system of Fig. 2. **b** Pressure stability gradient pump running through the nanotrap column and analytical column for three hours of measurements using the system of Fig. 2

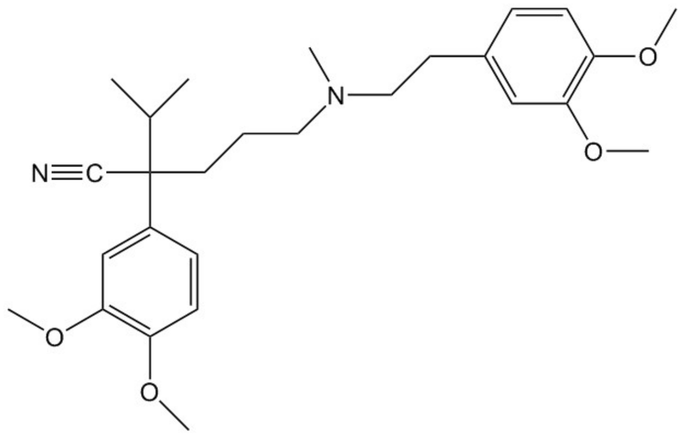

Fig. S3 Chemical structure of verapamil

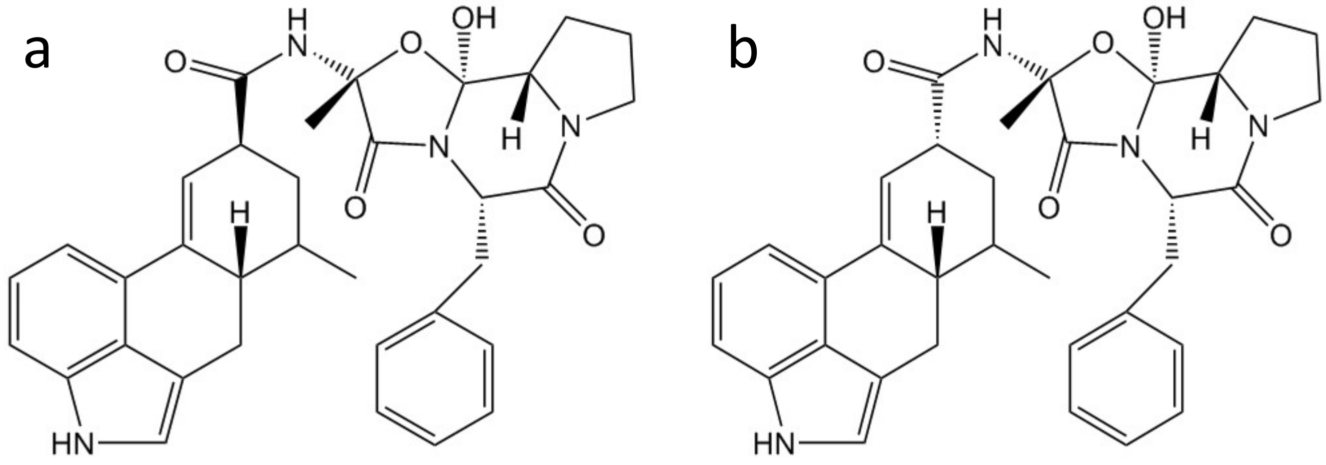

Fig. S4 Chemical structures of **a** ergotamine and **b** ergotaminine

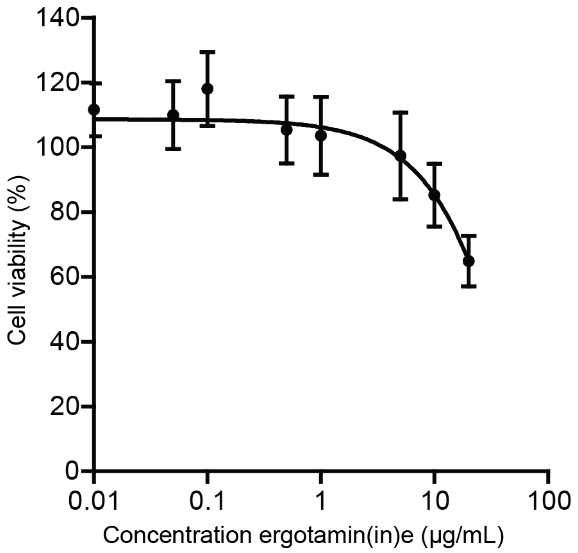

Fig. S5 Cell viability of a Caco-2/HT29-MTX-E12 co-culture after 24 h exposure to increasing concentrations of ergotamin(in)e using the WST-1 mitochondrial activity assay. Viability is given as a percentage of the control (% ± SEM; n=3)

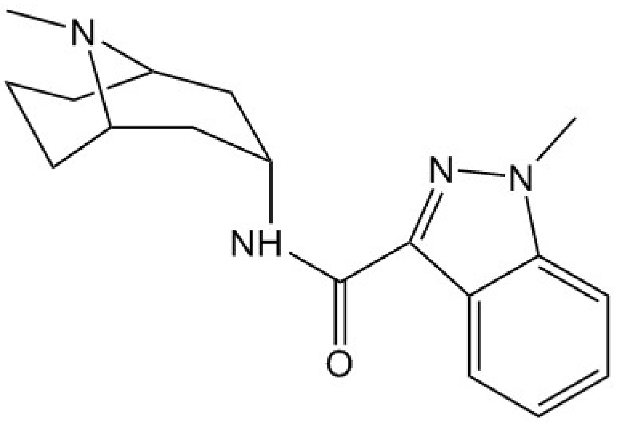

Fig. S6 Chemical structure granisetron

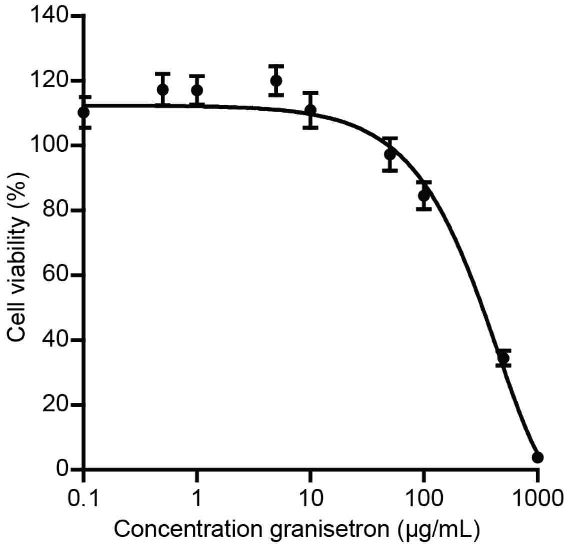

Fig. S7 Cell viability of a Caco-2/HT29-MTX-E12 co-culture after 24 h exposure to increasing concentrations of granisetron using the WST-1 mitochondrial activity assay. Viability is given as a percentage of the control (% ± SEM; n=3)

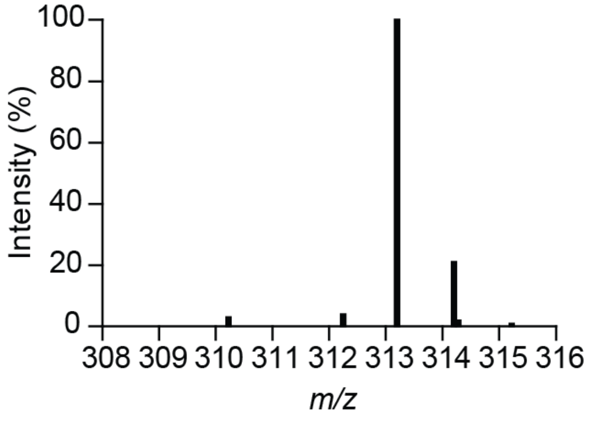

Fig. S8 Centroid spectrum of granisetron after 24 hour exposure to 37°C in a 24-well plate without cells

Supplemental Tables

Table S1 Multiple reaction monitoring (MRM) acquisition parameters for offline LC-MS/MS analysis of verapamil and ergotamin(in)e. The reconstructed ion current (RIC) of the transitions in bold were used for quantitation using external calibration.

Compound	Precursor (*m/z*)	Product (*m/z*)	Cone (V)	Collision energy (eV)
Verapamil	455.4	150.1	40	25
		**165.1**	40	25
		303.4	40	25
Ergotamin(in)e	582.4	**208.1**	30	40
		223.1	30	35
		268.1	30	25
		277.1	30	25

Table S2 Valve switching program for online coupling of dynamic flow through transwell effluents with chip-based UPLC-QTOFMS

Apical Sample			Basolateral Sample		
Time (min)	Switching valve	Action	Time (min)	Switching valve	Action
Initial	1	Off	Initial	1	On
Initial	2	On	Initial	2	Off
Initial	3	On	Initial	3	Off
0.1	1	On	0.1	1	Off
0.1	2	Off	0.1	2	On
4.10	3	Off	4.10	3	On

Supplemental Methods

Evaluation of carry-over in the online chip-based UPLC-QTOFMS analysis system

Carry-over for the online chip-based UPLC-QTOFMS analysis system was assessed for the initial (Fig. S1) and final (Fig. 2) systems by removing the flow through transwell model and instead connecting two syringes directly at the valve inlets for the apical and basolateral effluent. First, the syringe connected at the apical inlet contained 5 µg/mL of verapamil in HBSS and the syringe connected at the basolateral inlet contained HBSS only. The online analysis system was run for 6-8 measurements, and any appearance of a verapamil peak in the basolateral measurements was a sign of carry over. This experiment was repeated, but then with the syringe containing 5 µg/mL of verapamil attached at the basolateral valve inlet.
